# Effects of curcumin on low-density lipoprotein oxidation: From experimental studies to clinical practice

**DOI:** 10.17179/excli2022-4878

**Published:** 2022-06-22

**Authors:** Fatemeh Baratzadeh, Alexandra E. Butler, Prashant Kesharwani, Seyed Adel Moallem, Amirhossein Sahebkar

**Affiliations:** 1 School of Pharmacy, Mashhad University of Medical Sciences, Mashhad, Iran; 2 Department of Clinical Pharmacy, School of Pharmacy, Isfahan University of Medical Sciences, Isfahan, Iran; 3 Research Department, Royal College of Surgeons in Ireland, PO Box 15503, Adliya, Bahrain; 4 Department of Pharmaceutics, School of Pharmaceutical Education and Research, Jamia Hamdard, New Delhi, India; 5 Department of Pharmacology and Toxicology, College of Pharmacy, Al-Zahraa University for Women, Karbala, Iraq; 6 Department of Pharmacodynamics and Toxicology, School of Pharmacy, Mashhad University of Medical Sciences, Mashhad, Iran; 7 Applied Biomedical Research Center, Mashhad University of Medical Sciences, Mashhad, Iran; 8 Biotechnology Research Center, Pharmaceutical Technology Institute, Mashhad University of Medical Sciences, Mashhad, Iran; 9 School of Medicine, The University of Western Australia, Perth, Australia; 10 Department of Biotechnology, School of Pharmacy, Mashhad University of Medical Sciences, Mashhad, Iran

**Keywords:** oxidized low density lipoprotein, ox-LDL, anti-ox-LDL, Curcumin, Turmeric, Curcuma longa

## Abstract

Atherosclerosis is the most frequent cause of death globally. Oxidized low-density lipoprotein (ox-LDL) has an essential role in the formation of atherosclerotic plaques and foamy macrophages. Ox-LDL increases the uptake of cholesterol by macrophages and is the major cause of blood flow disruption. Ox-LDL is produced during oxidative stress and treatment with antioxidants could inhibit the production and function of ox-LDL. Curcumin is a potent antioxidant and has a strong track record in the treatment of numerous diseases. Recent studies indicate that Curcumin exerts a lipid-lowering effect, and can modulate the formation of atherosclerotic plaque. The current review focuses upon the role of Curcumin in oxidation of LDL and foam cell formation in atherosclerotic lesions.

## Introduction

### Oxidized low-density lipoprotein and atherosclerosis

According to a recent report, ischemic heart disease (IHD) remains the major cause of mortality worldwide (WHO, 2019[61]). Several lines of evidence indicate that atherosclerosis is the primary cause of IHD (Gimbrone et al., 2000[19]). Atherosclerosis is an inflammatory disease that occurs in the sub-endothelial space of medium and large-sized arteries at regions where laminar blood flow is disturbed (Wick et al., 2004[62]). The critical risk factor for atherosclerosis is dyslipidemia, defined as raised levels of low-density lipoprotein (LDL) and suppressed levels of high-density lipoprotein (HDL) (Garg et al., 2015[18]). In addition, the role of oxidized low-density lipoproteins (ox-LDL) in the development of atherosclerosis has been studied for many years (Quinn et al., 1987[45]; Parthasarathy et al., 1988[42]; Wang et al., 2020[60]). 

Conceptually, modified forms of LDL, but not native LDL, can induce cholesterol accumulation in monocytes or macrophages because the affinity of modified LDL to interact with scavenger receptors (SRs) of macrophages is greater than that of native LDL. The main type of modified LDL that can cause cholesterol accumulation is ox-LDL (Steinberg, 1997[55]; Yoshida and Kisugi, 2010[65]; Trpkovic et al., 2015[58]). Further evidence has illustrated that SRs, such as CD36 (also known as platelet glycoprotein IV), perform most of the ox-LDL uptake by macrophages, which is the first step of atherosclerosis that leads to the formation of foamy macrophages. Macrophages containing ox-LDL release a variety of inflammatory substances and cytokines. Moreover, ox-LDL (via CD36) traps macrophages by inhibiting cell migration, which consequently disrupts blood flow (Febbraio et al., 2001[14]; Steinberg and Witztum, 2010[56]; Park 2014[40]). 

**Figure 1:**
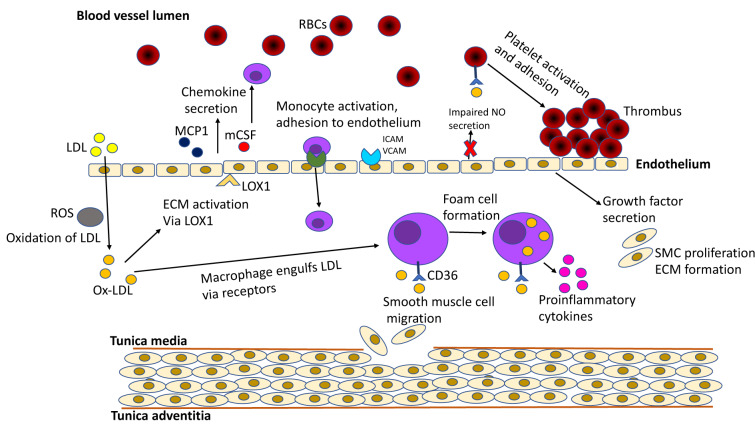
Summary of the role of Ox-LDL in atherosclerosis progression. Ox-LDL promotes atherosclerotic events right from their initiation in the subendothelium. Due to downregulated LDL receptors, the native LDL cannot be internalized by macrophages. Ox-LDL, via LOX 1 and other factors, activates endothelium for a number of events: adherence of LDL, monocytes, and platelets; secretion of chemokines and growth factors; production of ROS; impairing NO secretion. SRs, CD36, and LOX 1 help in the uptake of Ox-LDL by monocyte-derived macrophages in the subendothelium. Growth factors mediate SMC proliferation and extracellular matrix formation. Platelet adherence and accumulation is also, in part elicited by Ox-LDL, which results in a rupture-prone thrombus. Adapted from Khatana et al. (2020).

Ox-LDL is produced during oxidative stress. The physiological imbalance between pro-oxidant and antioxidant species can cause oxidative stress (Halliwell and Poulsen, 2006[22]). In addition, oxidative stress prepares endothelium to form oxidized cholesterol as a result of LDL oxidation (Yang et al., 2017[64]). During atherosclerosis, ox-LDL promotes the formation of atherosclerotic plaques (Mitra et al., 2011[33]). Ox-LDL performs this action through the lectin-like ox-LDL receptor 1 (LOX-1) (Pirillo et al., 2013[43]). Overexpression of LOX-1 in endothelial cells occurs in the process of atherosclerosis. In brief, ox-LDL stimulates atherosclerotic events, starting from disruption of the endothelial cells, reduced cholesterol efflux, foam cell formation, cytokine release from macrophages, and proliferation to platelet aggregation (Figure 1(Fig. 1)) (Ehara et al., 2001[12]; Khatana et al., 2020[24]). Thus, ox-LDL plays an essential role in atherosclerotic plaque formation and should be monitored and managed in patients with cardiovascular disease (CVD) risk factors. 

### Curcumin and atherosclerosis

Curcumin is obtained from the rhizomes of the *Curcuma longa *(Turmeric), a member of the ginger family. Curcumin is an important Asian golden spice and has an impressive impact on the taste and color of foods (Kocaadam and Şanlier, 2017[25]). Curcumin has a long history in the treatment of diseases, such as gastric and hepatic disorders, dental problems, menstrual difficulties, infectious diseases, malignancies, immune-related and metabolic disorders (Chaturvedi, 2009[8]; Sahebkar, 2010[46]; Gupta et al., 2013[21]; Panahi et al., 2014[39]; Sahebkar and Henrotin, 2016[47]; Panahi et al., 2017[39]; Bagherniya et al., 2018[3]; Parsamanesh et al., 2018[41]; Gorabi et al., 2019[20]; Mortezaee et al., 2019[37]; Shakeri et al., 2019[50]; Zahedipour et al., 2020[66]; Afshari et al., 2021[1]; Fu et al., 2021[15]; Mohammed et al., 2021[34]). Anti-inflammatory and antioxidant properties of Curcumin, a polyphenol, have been reported in numerous studies (Menon and Sudheer 2007[31]; Shehzad et al., 2011[51]; Momtazi-Borojeni et al., 2018[36]; Farhood et al., 2019[13]). Curcumin can improve the lipid profile of patients by reducing serum triglyceride (TG), LDL and total cholesterol levels significantly in subjects with coronary artery disease (CAD) (Qin et al., 2017[44]; Simental-Mendía et al., 2019[52]). Curcumin decreases the aortic lipid lesions and inhibits development of atherosclerotic plaques (Wongcharoen and Phrommintikul 2009[63]). Curcumin demonstrates antioxidant activity because the benzene rings in the structure of the Curcumin molecule eliminate reactive oxygen species (ROS) (Joe and Lokesh, 1994[23]). According to cardiac-related studies, serum levels of lipid peroxides are higher in patients with IHD; Curcumin is able to reduce lipid peroxide concentration (Stringer et al., 1989[57]; Soni and Kuttan 1992[53]). These actions could indicate that curcumin also inhibits ox-LDL elevation. However, the mechanisms underlying Curcumin's ability to effect improvement in atherosclerosis have not been investigated.

Based on the above findings, the current review aims to outline the known inhibitory effects of Curcumin on LDL oxidation and the formation of atherosclerotic plaque. Herein, we have reviewed the relevant preclinical and clinical studies. 

## Methods

The electronic databases PubMed, Embase, Google Scholar, and Scopus were searched using the following keywords from the beginning to December 2021 for preclinical and clinical trials on the effects of Curcumin in the oxidation of LDL or its inhibitory impact on Ox-LDL. The searched keywords were *oxidized low-density lipoprotein*, *oxLDL*, *ox-LDL*, *Curcumin*, *Turmeric* and, *Curcuma longa*. Inclusion criteria were clinical trials or animal studies that investigated the effects of Curcumin in atherosclerosis and full texts accessible on-line in English, with no limits of publishing date. Exclusion criteria were duplicated published materials and review articles. Data were collected between September 2021 and December 2021. In Tables 1(Tab. 1) (References in Table 1: Altinel et al., 2021[2]; Chen et al., 2015[9]; Kou et al., 2013[26]; Lee et al., 2010[27]; Min et al., 2013[32]), 2(Tab. 2) (References in Table 2: Altinel et al., 2021[2]; Um et al., 2014[59]) and 3(Tab. 3) (References in Table 3: Campos-Cervantes et al., 2011[7]; Funamoto et al., 2016[17], 2019[16]; Sahebkar et al., 2013[48]), we summarize the included studies.

## Results

### Curcumin use and LDL oxidation in cell studies

Experimental evidence concludes that Curcumin could modulate lipid transportation and prevent foam cell formation (Momtazi-Borojeni et al., 2019[37]). Altinel et al. investigated the effects of Curcumin on the histopathological changes in intestinal, vascular and lung tissues of rats post-splenectomy. In this experimental study, rats were randomly assigned into the following groups: only laparotomy group; only splenectomy group; Curcumin group that underwent splenectomy accompanied by 20 mg/kg Curcumin; corn oil group that underwent splenectomy accompanied by 20 mg/kg corn oil for 28 days. After sacrifice, an aortic segment, distal segments of total colectomy specimen, and lung segments were collected for histopathological examination. Histopathological analysis of vascular tissues from the 4 groups showed no foamy macrophages in the vascular wall, with no significant difference between the groups (Altinel et al., 2021[2]).

Macrophages are an essential contributor to the development and stability of atherosclerotic plaques. M1 macrophages induce a proinflammatory response and are present in unstable plaques (Biswas et al., 2012[5]; Cho et al., 2013[10]). Since the role of Curcumin in modulating the uptake of cholesterol in M1 macrophages had not been well studied, Chen et al. investigated the effect of Curcumin on foam cell formation in M1 macrophages stimulated with ox-LDL. In this study, M1 macrophages were incubated with 6.25 and 12.5 mmol/L of Curcumin for 12 h. Moreover, to determine the effects of Curcumin on foam cell formation, M0 and the treated M1 macrophages were incubated with 30 mg/L oxLDL for 24 h with or without the peroxisome proliferator-activated receptor gamma (PPARγ) inhibitor, as PPARγ has a direct effect on the presentation of CD36 in macrophages (Lim et al., 2006[29]). The results showed that 6.25 and 12.5 mmol/L curcumin increased foam cell formation induced by ox-LDL. Curcumin, dose-dependently, also increased CD36 and PPARγ levels in the ox-LDL-induced M1 cells (Chen et al., 2015[9]). These two reviewed studies have not shown a beneficial effect of curcumin in reducing ox-LDL.

Lipid accumulation in macrophages is mediated by several SRs, such as CD36, and is the initial step in atherosclerosis (Collot-Teixeira et al., 2007[11]). LOX-1 has been identified as a major SR for ox-LDL in endothelial cells and, further, LOX-1 gene expression is induced by elevated ox-LDL levels (Sawamura et al., 1997[49]; Li and Mehta, 2000[28]). Kou et al. investigated the effects of 1-50 µM of Curcumin upon the expression of SRs in THP-1 macrophages and monocytes. In this experiment, LDL was oxidized with Cu^2+^. The results showed that Curcumin could induce CD36 expression. Curcumin also dose-dependently inhibited the uptake of ox-LDL and reduced the expression of CD36 surface protein (using 20 µM Curcumin). The authors suggested that Curcumin could control the expression of CD36 at different levels, including transcription, translation, and translocation. THP-1 monocytes treated with Curcumin showed a significant dose-dependent suppression of LOX-1 mRNA expression (Kou et al., 2013[26]).

Lee et al. evaluated the inhibitory effects of Curcumin on TNFα-induced LOX-1 expression in human umbilical vein endothelial cells (HUVECs). In this experiment, HUVECs were pre-treated with Curcumin for 1 hour before incubation with TNFα for 12 hours. Incubation of cells with TNFα increases the expression of LOX-1. The results showed that 1, 5, 10, and 25 μg/mL of Curcumin dose-dependently suppressed the production of ROS and LOX-1 expression in HUVECs. The authors suggested that Curcumin could be beneficial in preventing atherosclerosis (Lee et al., 2010[27]).

Min et al. investigated the effects of Curcumin on the formation of foamy macrophages and the expression of SRs in RAW 264.7 macrophage cell lines. Murine macrophages were treated with native LDL or ox-LDL with or without Curcumin for 24 h, and the lipid accumulation by macrophages was measured. The results showed that Curcumin inhibits PPARγ and CD36 expression. Curcumin also inhibited ox-LDL-induced foam cell formation (Min et al., 2013[32]). 

### Curcumin use and LDL oxidation in animal studies

A limited number of animal studies have been conducted to examine the effects of Curcumin on blood levels of ox-LDL. Altinel et al. investigated the effects of Curcumin on lipid profile and oxidative markers in rats after post-splenectomy. In this experiment, rats were randomly assigned into 4 groups: only laparotomy Group; only splenectomy Group; Curcumin group that underwent splenectomy with 20 mg/kg curcumin; corn oil group underwent splenectomy with 20 mg/kg corn oil for 28 days. Blood samples were collected at time of sacrifice. The primary findings indicated that blood levels of ox-LDL and LOX-1, which were elevated in the splenectomy group, were significantly reduced by a single dose of Curcumin (Altinel et al., 2021[2]). 

Um et al. investigated the effects of Curcumin supplementation on inhibiting atherosclerosis in white rabbits (Um et al., 2014[59]). The white rabbits were divided into 3 groups in a random manner receiving regular chow diet, a regular chow diet with 1 % cholesterol, or a regular chow diet with 1 % cholesterol plus 0.2 % curcumin for 8 weeks. All rabbits were sacrificed, and blood samples were collected from the retro-orbital sinus. Findings of this study showed that Curcumin significantly reduced blood levels of ox-LDL. The authors suggested that curcumin could prevent progression of atherosclerosis.

### Curcumin use and LDL oxidation in clinical studies

Campos-Cervantes et al. in a prospective, randomized, simple-blind, controlled study, determined the effect of curcumin on ox-LDL levels of obese patients. In this clinical trial, participants were divided into 3 groups: two groups received a daily dose of 500 and 750 mg of Curcumin, respectively, whilst the third group received placebo for 12 weeks. The plasma levels of ox-LDL were determined at 0, 2, 6, and 12 weeks. The authors reported that 500 mg of Curcumin significantly decreased the ox-LDL levels at 2, 6, and 12 weeks. However, 750 mg of Curcumin did not significantly reduce the serum levels of ox-LDL (Campos-Cervantes et al., 2011[7]).

Recently, α1-Antitrypsin-low density lipoprotein (AT-LDL) was identified as a form of ox-LDL. AT-LDL can cause atherogenesis and is found at the site of atherosclerotic plaques. Serum levels of AT-LDL indicate the activity of foam cells in atherosclerotic plaques (Mashiba et al., 2001[30]). Funamoto et al., in a randomized, double blind parallel group study, examined the effects of Curcumin on the level of plasma ox-LDL in patients with non-insulin-dependent diabetes mellitus. Patients were divided into two groups in a random manner. The first group received 180 mg/day of curcumin for 6 months, and the second group was considered as placebo. Serum levels of AT-LDL were measured at 0, 3 and, 6 months after the intervention. This clinical trial showed that Curcumin inhibited the increase in plasma levels of AT-LDL. By comparison, the plasma level of AT-LDL in the placebo group increased significantly (Funamoto et al., 2019[16]). 

Funamoto et al. also examined the effects of Curcumin on the level of AT-LDL in subjects with mild-moderate chronic obstructive pulmonary disease (COPD). The authors conducted a randomized, double blind, parallel-group study. Subjects were randomly assigned to receive 180 mg/day of Curcumin or placebo for 24 weeks, and changes in serum AT-LDL were evaluated. The results showed a significantly lower AT-LDL level in the Curcumin group (Funamoto et al., 2016[17]). 

Curcuminoids are anti-atherogenic agents because of their antioxidant, antithrombotic and anti-inflammatory actions (Srivastava et al., 1985[54]; Basnet and Skalko-Basnet, 2011[4]). Furthermore, autoantibodies to ox-LDL have been found in atherosclerotic plaques and in the plasma of atherosclerosis patients (Bui et al., 1996[6]). Sahebkar et al., in a randomized, double blind, cross-over study, examined the effects of Curcuminoids on the level of serum anti-ox-LDL in obese individuals. The subjects were divided into two groups to receive 1 g/day of Curcuminoids or placebo for 30 days. After a 2-week washout period, subjects received the alternate treatment. Blood samples were collected from participants before and after each dietary treatment phase. At the end of the 10^th^ week, no significant change was observed in serum levels of anti-ox-LDL (Sahebkar et al., 2013[48]).

## Conclusion

Dyslipidemia is one of the main causes of atherosclerosis, resulting in CVD and IHD. Lipid disorders increase with age and impact many lives globally. Recent research has concluded that ox-LDL is a novel biomarker for the development of dyslipidemia and atherosclerosis. Ox-LDL is produced during oxidative stress conditions and has a major role in the formation of atherosclerotic plaques and foamy macrophages. The use of antioxidant therapy in reducing oxidative stress or ox-LDL has been examined in several articles. However, insufficient evidence currently exists to use them therapeutically to prevent atherosclerotic plaque. Herbal medicines have great potential for reducing free radicals and are currently used in numerous clinical trials as antioxidants to combat oxidative stress. Curcumin, as an herbal antioxidant, has shown beneficial effects in improving lipid disorders and cardiovascular events in several clinical and animal studies. Proposed mechanisms for curcumin's action in reducing atherosclerotic plaques include factors such as reduction of blood lipid levels and ox-LDL production, decrease in macrophage and inflammatory cytokine migration to the vessel wall, a reduction in expression of specific ox-LDL receptors such as CD36 and LOX-1, a reduction in the uptake of cholesterol and inhibition of foamy macrophage production. 

**Figure 2:**
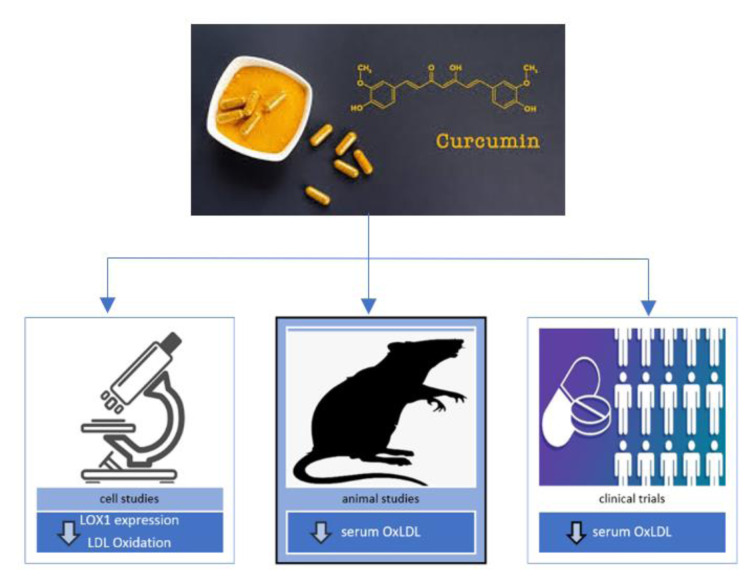
The positive effects of Curcumin on Ox-LDL

In this article, we have reviewed the effects of Curcumin on the oxidation of LDL and the formation of atherosclerotic plaques. Preclinical studies performed in cell lines gave contradictory results. Among the five preclinical studies reviewed, two of them showed that Curcumin reduced LDL oxidation and decreased the expression of LOX-1; however, the effects of curcumin on reducing foam cell formation, uptake of ox-LDL, and expression of CD36 and PPARγ were not the same in these two preclinical studies. Both animal studies that investigated the effect of Curcumin on blood levels of ox-LDL showed that Curcumin could reduce plasma levels of ox-LDL. One animal experiment showed that Curcumin could also reduce plasma levels of LOX-1. Four clinical trials that we reviewed here were randomized and had a control group. These studies investigated the effect of Curcumin on plasma levels of ox-LDL, AT-LDL and autoantibodies against ox-LDL. Curcumin decreased plasma levels of ox-LDL and AT-LDL, but no significant change was observed in circulating anti-ox-LDLs. The positive effects of curcumin on ox-LDL are summarized in Figure 2(Fig. 2).

Overall, our results suggest that curcumin could be effective in reducing LDL oxidation. However, additional investigations are necessary to definitively determine whether Curcumin could serve as standard therapy for the prevention of atherosclerosis, both as an adjunct and a monotherapy.

## Declaration

### Funding

There is no funding for this research.

### Conflict of interest

None.

## Figures and Tables

**Table 1:**
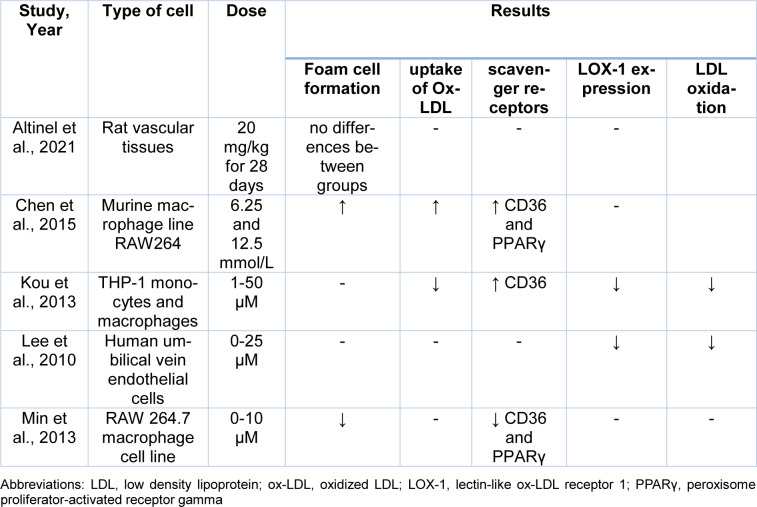
Effects of curcumin on LDL oxidation in pre-clinical studies

**Table 2:**
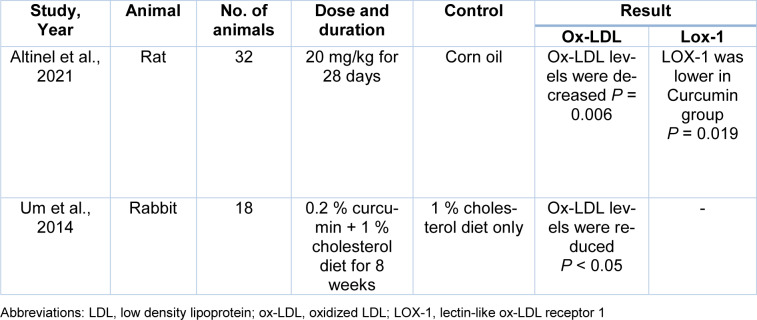
Effects of curcumin on circulating oxidized LDL levels in pre-clinical studies

**Table 3:**
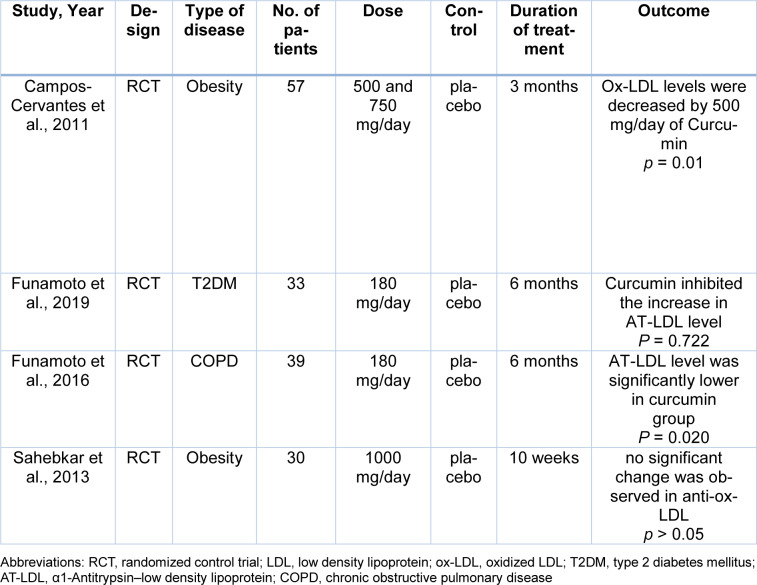
Effects of curcumin on oxidized LDL in clinical studies

## References

[1] Afshari AR, Jalili-Nik M, Abbasinezhad-Moud F, Javid H, Karimi M, Mollazadeh H (2021). Anti-tumor effects of curcuminoids in glioblastoma multiforme: An updated literature review. Curr Med Chem.

[2] Altinel Y, Gulcicek OB, Kose E, Karacaglar A, Demirgan S, Sozer V (2021). Systemic amelioration via curcumin in rats following splenectomy: lipid profile, endothelial and oxidative damage. J Invest Surg.

[3] Bagherniya M, Nobili V, Blesso CN, Sahebkar A (2018). Medicinal plants and bioactive natural compounds in the treatment of non-alcoholic fatty liver disease: A clinical review. Pharmacol Res.

[4] Basnet P, Skalko-Basnet N (2011). Curcumin: an anti-inflammatory molecule from a curry spice on the path to cancer treatment. Molecules.

[5] Biswas SK, Chittezhath M, Shalova IN, Lim JY (2012). Macrophage polarization and plasticity in health and disease. Immunol Res.

[6] Bui MN, Sack MN, Moutsatsos G, Lu DY, Katz P, McCown R (1996). Autoantibody titers to oxidized low-density lipoprotein in patients with coronary atherosclerosis. Am Heart J.

[7] Campos-Cervantes A, Alvarado-Caudillo Y, Perez-Vazquez V, Ramirez- Emiliano J, Murillo-Ortiz BO (2011). Curcumin decreases the oxidative damage indexes and increases the adiponectin levels in serum of obese subjects. Free Rad Biol Med.

[8] Chaturvedi T (2009). Uses of turmeric in dentistry: An update. Indian J Dent Res.

[9] Chen F-Y, Zhou J, Guo N, Ma W-G, Huang X, Wang H (2015). Curcumin retunes cholesterol transport homeostasis and inflammation response in M1 macrophage to prevent atherosclerosis. Biochem Biophys Res Commun.

[10] Cho KY, Miyoshi H, Kuroda S, Yasuda H, Kamiyama K, Nakagawara J (2013). The phenotype of infiltrating macrophages influences arteriosclerotic plaque vulnerability in the carotid artery. J Stroke Cerebrovasc Dis.

[11] Collot-Teixeira S, Martin J, McDermott-Roe C, Poston R, McGregor JL (2007). CD36 and macrophages in atherosclerosis. Cardiovasc Res.

[12] Ehara S, Ueda M, Naruko T, Haze K, Itoh A, Otsuka M (2001). Elevated levels of oxidized low density lipoprotein show a positive relationship with the severity of acute coronary syndromes. Circulation.

[13] Farhood B, Mortezaee K, Goradel NH, Khanlarkhani N, Salehi E, Nashtaei MS (2019). Curcumin as an anti-inflammatory agent: Implications to radiotherapy and chemotherapy. J Cell Physiol.

[14] Febbraio M, Hajjar DP, Silverstein RL (2001). CD36: a class B scavenger receptor involved in angiogenesis, atherosclerosis, inflammation, and lipid metabolism. J Clin Invest.

[15] Fu YS, Chen TH, Weng L, Huang L, Lai D, Weng CF (2021). Pharmacological properties and underlying mechanisms of curcumin and prospects in medicinal potential. Biomed Pharmacother.

[16] Funamoto M, Shimizu K, Sunagawa Y, Katanasaka Y, Miyazaki Y, Hasegawa K (2019). Effects of highly absorbable curcumin in patients with impaired glucose tolerance and non-insulin-dependent diabetes mellitus. J Diabetes Res.

[17] Funamoto M, Sunagawa Y, Katanasaka Y, Miyazaki Y, Imaizumi A, Kakeya H (2016). Highly absorptive curcumin reduces serum atherosclerotic low-density lipoprotein levels in patients with mild COPD. Int J Chron Obstruct Pulmon Dis.

[18] Garg R, Aggarwal S, Kumar R, Sharma G (2015). Association of atherosclerosis with dyslipidemia and co-morbid conditions: A descriptive study. J Nat Sci Biol Med.

[19] Gimbrone MA, Topper JN, Nagel T, Anderson KR, Garcia-Cardeña G (2000). Endothelial dysfunction, hemodynamic forces, and atherogenesis. Ann N Y Acad Sci.

[20] Gorabi AM, Kiaie N, Hajighasemi S, Jamialahmadi T, Majeed M, Sahebkar A (2019). The effect of curcumin on the differentiation of mesenchymal stem cells into mesodermal lineage. Molecules.

[21] Gupta SC, Sung B, Kim JH, Prasad S, Li S, Aggarwal BB (2013). Multitargeting by turmeric, the golden spice: From kitchen to clinic. Mol Nutr Food Res.

[22] Halliwell BB, Poulsen HE, Halliwell BB, Poulsen HE (2006). Oxidative stress. Cigarette smoke and oxidative stress.

[23] Joe B, Lokesh BR (1994). Role of capsaicin, curcumin and dietary n-3 fatty acids in lowering the generation of reactive oxygen species in rat peritoneal macrophages. Biochim Biophys Acta.

[24] Khatana C, Saini NK, Chakrabarti S, Saini V, Sharma A, Saini RV (2020). Mechanistic insights into the oxidized low-density lipoprotein-induced atherosclerosis. Oxid Med Cell Longev.

[25] Kocaadam B, Şanlier N (2017). Curcumin, an active component of turmeric (Curcuma longa), and its effects on health. Crit Rev Food Sci Nutr.

[26] Kou M-C, Weng C-Y, Wu M-J, Chiou S-Y, Wang L, Ho C-T (2013). Curcuminoids distinctly exhibit antioxidant activities and regulate expression of scavenger receptors and heme oxygenase-1. Mol Nutr Food Res.

[27] Lee H-S, Lee M-J, Kim H, Choi S-K, Kim J-E, Moon H-I (2010). Curcumin inhibits TNFalpha-induced lectin-like oxidised LDL receptor-1 (LOX-1) expression and suppresses the inflammatory response in human umbilical vein endothelial cells (HUVECs) by an antioxidant mechanism. J Enzyme Inhib Med Chem.

[28] Li D, Mehta JL (2000). Upregulation of endothelial receptor for oxidized LDL (LOX-1) by oxidized LDL and implications in apoptosis of human coronary artery endothelial cells: evidence from use of antisense LOX-1 mRNA and chemical inhibitors. Arterioscler Thromb Vasc Biol.

[29] Lim H-J, Lee S, Lee K-S, Park J-H, Jang Y, Lee EJ (2006). PPARγ activation induces CD36 expression and stimulates foam cell like changes in rVSMCs. Prostaglandins Other Lipid Mediat.

[30] Mashiba S, Wada Y, Takeya M, Sugiyama A, Hamakubo T, Nakamura A (2001). In vivo complex formation of oxidized α1-antitrypsin and LDL. Arterioscler Thromb Vasc Biol.

[31] Menon VP, Sudheer AR (2007). Antioxidant and anti-inflammatory properties of curcumin. Adv Exp Med Biol.

[32] Min K-J, Um HJ, Kwon TK, Cho K-H (2013). Curcumin inhibits oxLDL-induced CD36 expression and foam cell formation through the inhibition of p38 MAPK phosphorylation. Food Chem Toxicol.

[33] Mitra S, Goyal T, Mehta JL (2011). Oxidized LDL, LOX-1 and atherosclerosis. Cardiovascr Drugs Ther.

[34] Mohammed ES, El-Beih NM, El-Hussieny EA, El-Ahwany E, Hassan M, Zoheiry M (2021). Effects of free and nanoparticulate curcumin on chemically induced liver carcinoma in an animal model. Arch Med Sci.

[35] Momtazi-Borojeni AA, Abdollahi E, Nikfar B, Chaichian S, Ekhlasi-Hundrieser M (2019). Curcumin as a potential modulator of M1 and M2 macrophages: new insights in atherosclerosis therapy. Heart Fail Rev.

[36] Momtazi-Borojeni AA, Haftcheshmeh SM, Esmaeili SA, Johnston TP, Abdollahi E, Sahebkar A (2018). Curcumin: A natural modulator of immune cells in systemic lupus erythematosus. Autoimmun Rev.

[37] Mortezaee K, Salehi E, Mirtavoos-mahyari H, Motevaseli E, Najafi M, Farhood B (2019). Mechanisms of apoptosis modulation by curcumin: Implications for cancer therapy. J Cell Physiol.

[38] Panahi Y, Ghanei M, Bashiri S, Hajihashemi A, Sahebkar A (2014). Short-term curcuminoid supplementation for chronic pulmonary complications due to sulfur mustard intoxication: positive results of a randomized double-blind placebo-controlled trial. Drug Res.

[39] Panahi Y, Khalili N, Sahebi E, Namazi S, Reiner Ž, Majeed M (2017). Curcuminoids modify lipid profile in type 2 diabetes mellitus: A randomized controlled trial. Complement Ther Med.

[40] Park YM (2014). CD36, a scavenger receptor implicated in atherosclerosis. Exp Mol Med.

[41] Parsamanesh N, Moossavi M, Bahrami A, Butler AE, Sahebkar A (2018). Therapeutic potential of curcumin in diabetic complications. Pharm Res.

[42] Parthasarathy S, Quinn MT, Steinberg D (1988). Is oxidized low density lipoprotein involved in the recruitment and retention of monocyte/macrophages in the artery wall during the initiation of atherosclerosis?. Basic Life Sci.

[43] Pirillo A, Norata GD, Catapano AL (2013). LOX-1, OxLDL, and atherosclerosis. Mediators Inflamm.

[44] Qin S, Huang L, Gong J, Shen S, Huang J, Ren H (2017). Efficacy and safety of turmeric and curcumin in lowering blood lipid levels in patients with cardiovascular risk factors: a meta-analysis of randomized controlled trials. Nutr J.

[45] Quinn MT, Parthasarathy S, Fong LG, Steinberg D (1987). Oxidatively modified low density lipoproteins: a potential role in recruitment and retention of monocyte/macrophages during atherogenesis. Proc Natl Acad Sci.

[46] Sahebkar A (2010). Molecular mechanisms for curcumin benefits against ischemic injury. Fertil Steril.

[47] Sahebkar A, Henrotin Y (2016). Analgesic efficacy and safety of curcuminoids in clinical practice: A systematic review and meta-analysis of randomized controlled trials. Pain Med (United States).

[48] Sahebkar A, Mohammadi A, Atabati A, Rahiman S, Tavallaie S, Iranshahi M (2013). Curcuminoids modulate pro-oxidant-antioxidant balance but not the immune response to heat shock protein 27 and oxidized LDL in obese individuals. Phytother Res.

[49] Sawamura T, Kume N, Aoyama T, Moriwaki H, Hoshikawa H, Aiba Y (1997). An endothelial receptor for oxidized low-density lipoprotein. Nature.

[50] Shakeri A, Cicero AFG, Panahi Y, Mohajeri M, Sahebkar A (2019). Curcumin: A naturally occurring autophagy modulator. J Cell Physiol.

[51] Shehzad A, Ha T, Lee YS, Subhan F (2011). New mechanisms and the anti-inflammatory role of curcumin in obesity and obesity-related metabolic diseases. Eur J Nutr.

[52] Simental-Mendía LE, Pirro M, Gotto AM, Banach M, Atkin SL, Majeed M (2019). Lipid-modifying activity of curcuminoids: A systematic review and meta-analysis of randomized controlled trials. Crit Rev Food Sci Nutr.

[53] Soni KB, Kuttan R (1992). Effecf of oral curcumin administration on serum peroxides and cholesterol levels in human volunteers. Indian J Physiol Pharmacol.

[54] Srivastava R, Dikshit M, Srimal R, Dhawan B (1985). Anti-thrombotic effect of curcumin. Thrombosis Res.

[55] Steinberg D (1997). Lewis A. Conner Memorial Lecture: Oxidative modification of LDL and atherogenesis. Circulation.

[56] Steinberg D, Witztum JL (2010). Oxidized low-density lipoprotein and atherosclerosis. Arterioscleros Thromb Vasc Biol.

[57] Stringer MD, Görög PG, Freeman A, Kakkar VV (1989). Lipid peroxides and atherosclerosis. Brit Med J.

[58] Trpkovic A, Resanovic I, Stanimirovic J, Radak D, Mousa SA, Cenic-Milosevic D (2015). Oxidized low-density lipoprotein as a biomarker of cardiovascular diseases. Crit Rev Clin Lab Sci.

[59] Um MY, Hwang KH, Choi WH, Ahn J, Jung CH, Ha TY (2014). Curcumin attenuates adhesion molecules and matrix metalloproteinase expression in hypercholesterolemic rabbits. Nutr Res.

[60] Wang A, Dai L, Zhang N, Lin J, Chen G, Zuo Y (2020). Oxidized low-density lipoprotein (LDL) and LDL cholesterol are associated with outcomes of minor stroke and TIA. Atherosclerosis.

[61] WHO, World Health Organization Global Health Estimates. https://www.who.int/data/gho/data/themes/mortality-and-global-health-estimates.

[62] Wick G, Knoflach M, Xu Q (2004). Autoimmune and inflammatory mechanisms in atherosclerosis. Annu Rev Immunol.

[63] Wongcharoen W, Phrommintikul A (2009). The protective role of curcumin in cardiovascular diseases. Int J Cardiol.

[64] Yang X, Li Y, Li Y, Ren X, Zhang X, Hu D (2017). Oxidative stress-mediated atherosclerosis: mechanisms and therapies. Front Physiol.

[65] Yoshida H, Kisugi R (2010). Mechanisms of LDL oxidation. Clin Chim Acta.

[66] Zahedipour F, Hosseini SA, Sathyapalan T, Majeed M, Jamialahmadi T, Al-Rasadi K (2020). Potential effects of curcumin in the treatment of COVID-19 infection. Phytother Res.

